# Hydroxytyrosol Benefits Boar Semen Quality via Improving Gut Microbiota and Blood Metabolome

**DOI:** 10.3389/fnut.2021.815922

**Published:** 2022-01-17

**Authors:** Hui Han, Ruqing Zhong, Yexun Zhou, Bohui Xiong, Liang Chen, Yue Jiang, Lei Liu, Haiqing Sun, Jiajian Tan, Fuping Tao, Yong Zhao, Hongfu Zhang

**Affiliations:** ^1^State Key Laboratory of Animal Nutrition, Institute of Animal Sciences, Chinese Academy of Agricultural Sciences, Beijing, China; ^2^Precision Livestock and Nutrition Unit, Gembloux Agro-Bio Tech, University of Liège, Gembloux, Belgium; ^3^YangXiang Joint Stock Company, Guigang, China; ^4^Hangzhou Viablife Biotech Co., Ltd., Hangzhou, China

**Keywords:** hydroxytyrosol, boar, semen quality, gut microbiota, blood metabolome

## Abstract

Semen quality is one of the most important factors for the success of artificial insemination which has been widely applied in swine industry to take the advantages of the superior genetic background and higher fertility capability of boars. Hydroxytyrosol (HT), a polyphenol, has attracted broad interest due to its strong antioxidant, anti-inflammatory, and antibacterial activities. Sperm plasma membrane contains a large proportion of polyunsaturated fatty acids which is easily impaired by oxidative stress and thus to diminish semen quality. In current investigation, we aimed to explore the effects of dietary supplementation of HT on boar semen quality and the underlying mechanisms. Dietary supplementation of HT tended to increase sperm motility and semen volume/ejaculation. And the follow-up 2 months (without HT, just basal diet), the semen volume was significantly more while the abnormal sperm was less in HT group than that in control group. HT increased the “beneficial microbes” *Bifidobacterium, Lactobacillus, Eubacterium, Intestinimonas, Coprococcus, and Butyricicoccus*, however, decreased the relative abundance of “harmful microbes” *Streptococcus, Oscillibacter, Clostridium_sensu_stricto, Escherichia, Phascolarctobacterium*, and *Barnesiella*. Furthermore, HT increased plamsa steroid hormones such as testosterone and its derivatives, and antioxidant molecules while decreased bile acids and the derivatives. All the data suggest that HT improves gut microbiota to benefit plasma metabolites then to enhance spermatogenesis and semen quality. HT may be used as dietary additive to enhance boar semen quality in swine industry.

## Introduction

Artificial insemination (AI) has been extensively used in swine industry in order to take the advantages of the superior genetic background and higher fertility capability of boars and sows (1–4). The application of AI is restricted to the semen quality, which can be influenced by multiple factors including the boar itself (e.g., age) and the environment factors (e.g., temperature and light) (1–4). Sperm plasma membrane contains a large proportion of polyunsaturated fatty acids (PUFA), which is easily impaired by oxidative stress and thus to diminish semen quality (1, 2). Increasing evidence suggests the potential to use nutritional interventions to enhance antioxidant capacity for improving boar semen quality (3, 4). *In vitro* studies found that supplementation with exogenous antioxidants, such as rosmarinic acid, polysaccharide, and skim milk could improve boar sperm quality by alleviating oxidative stress during the cryopreservation (5–7). Moreover, a lot of evidence from *in vivo* studies also illustrated that dietary supplementation with antioxidants improved the boar semen quality. For example, dietary supplementation of L-arginine and lysine improved boar semen quality by increasing antioxidant capacity (3, 4).

Hydroxytyrosol (HT), a polyphenol, is extracted from olive leaves and oil. HT has attracted broad interest due to its strong antioxidant, anti-inflammatory, and antibacterial activities (8, 9). HT exhibited antioxidant capacity by scavenging oxidant chemical species and promoting the expressions of antioxidant enzymes via activating nuclear factor E2-related factor 2 (Nrf2) signaling (8). Furthermore, Wei et al. reported that HT increased bacterial membrane permeabilization and then interacted with DNA, which collectively inhibited bacterial growth (10). Our recent study also showed that olive fruit extracts enriching HT exhibited strong antioxidant effects by benefiting gut microbiota in mice (11). Moreover, our previous study found that improved gut microbiota was able to ameliorate sperm quality by modulating the plasma metabolomes and small intestinal function in mice (12, 13). To search the effective strategies to improve sperm quality and enhance the production of swine production, current investigation was designed to explore the effects of dietary supplementation of HT on the boar semen quality and the underlying mechanisms.

## Materials and Methods

### Boars and Experimental Design

**Figure 1:**
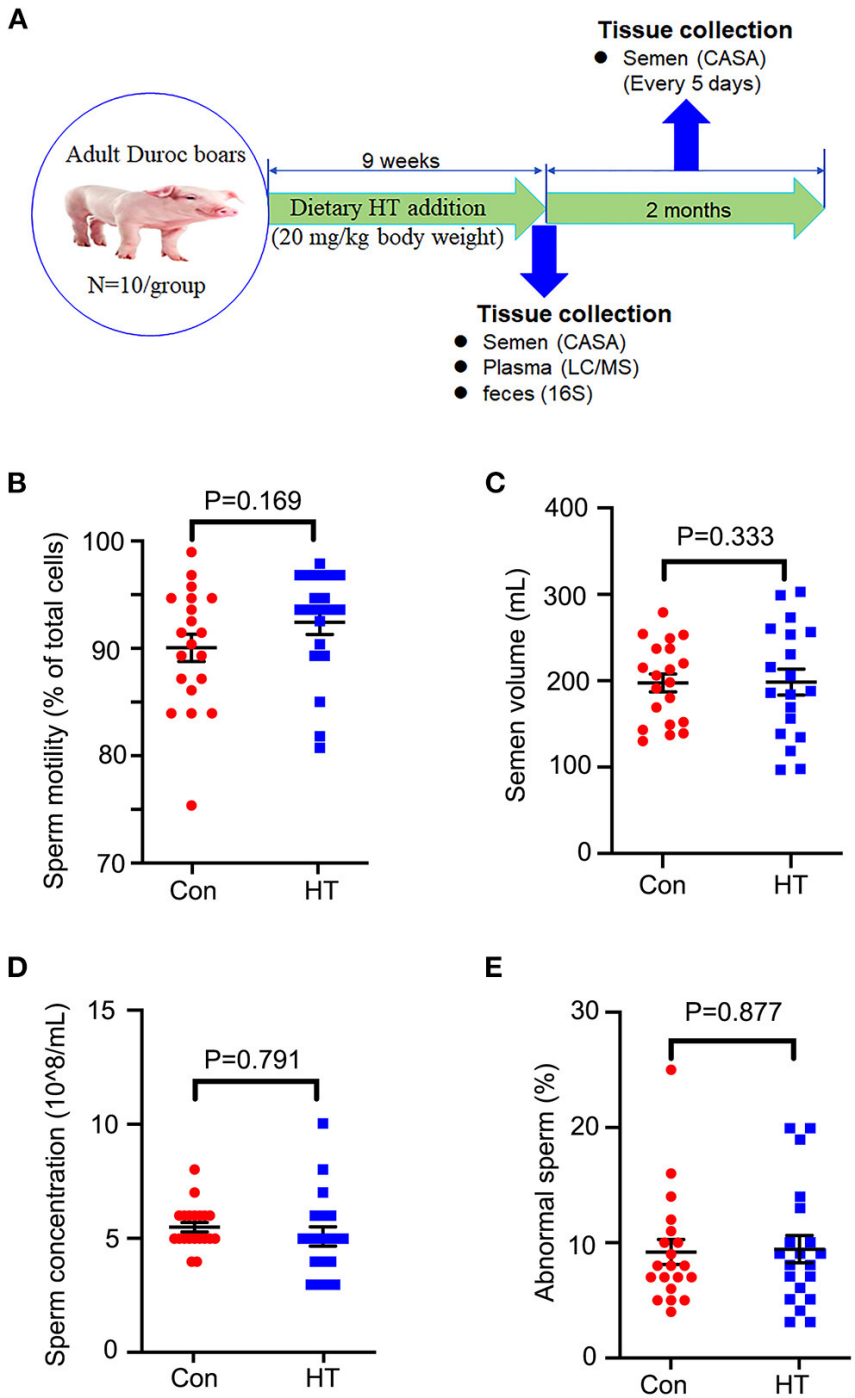
Effects of HT on the semen quality. **(A)** Study design. **(B)** Sperm motility. **(C)** Semen volume. **(D)** Sperm concentration. **(E)** Abnormal sperm. Data were expressed as the mean ± SEM.

All animal procedures were approved by the Animal Care and Use Committee of the Institute of Animal Sciences of Chinese Academy of Agricultural Sciences (IAS2021-67). A total of 20 healthy boars (Duroc) aged from 31 to 33 months were selected in this study at the artificial insemination center of Yangxiang Joint Stock Company (Guangxi, China) (1). Boar feeding conditions have been previously reported (2). All boars were randomly divided into 2 groups (*n* = 10 per group). Boars in the control group (Con) were fed with a commercially prepared corn and soybean meal-based diet (Table 1), and boars in the HT supplement group (HT) were fed with a basal diet supplemented with 20 mg/kg body weight of HT. The dose of HT was adopted according to our previous mice experiment (11). The boars received two meals at 11:00 and 17:00 and the total feed intake amounts were 2.5 kg (1.25 kg per meal) every day. HT was mixed with the diet when the boars received the first meal and HT was taken almost completely by the boars each day. HT (purity > 99%) was kindly donated by Viablife Biotech Co., Ltd. (Hangzhou, China). The boars were housed in individual crates and HT was supplied for 60 days (Figure 1A).

**Table 1:** Composition and nutrient analysis of basal diet.

**Ingredient**	**Content, %**
Corn	35.15
Barley	24.83
wheat	15.82
Rice bran meal	9.40
Soybean meal	7.90
Soybean oil	2.00
L-lysine	0.40
Methionine	0.14
Threonine	0.24
Ground limestone	1.44
Monocalcium phosphate	1.21
Sodium chloride	0.48
Premix^*^	1.00
Total	100
**Nutrient, %**	
Calculated NE, kcal/kg	2.24
Crude protein, %	14.50
Crude fat, %	3.22
Crude ash, %	6.18
Crude fiber, %	4.15

* *Premix provided the following minerals per kilogram: 17 mg Cu, 160 mg Fe, 140 mg Zn, 50 mg Mn, 0.50 mg I, 0.50 mg Se, and 0.22 mg Cr*.

Semen samples were collected by gloved-hand techniques. After collection, four semen parameters were assessed: semen volume, sperm concentration, sperm motility, and abnormal sperm rate, according to the reported methods (1, 14). Blood samples were collected into ethylenediamine tetraacetic acid (EDTA) plasma tubes by venipuncture from the hindlimb vein of boars during ejaculations. Each blood sample was then centrifuged at 3,000 × g for 10 min at 4°C to obtain a plasma sample and subsequently stored at −80°C until analysis. Each boar's rectum was massaged to stimulate defecation, and then fresh feces were collected and stored at −80°C for subsequent microbiota analysis (1).

The follow-up long term beneficial effects of HT on boar semen quality were determined. After HT supplementation, all the boars were fed with basal diet (without HT supplementation). The semen was collected every 5 days and the semen quality was analyzed. The long-term analysis was for 2 months (Figure 1A).

### Evaluation of Spermatozoa Motility Using a Computer-Assisted Sperm Analysis System

Spermatozoa motility and concentration, and abnormal sperm rate were determined by the computer-assisted sperm assay (CASA) method according to World Health Organization guidelines and our previous reports (ML-210JZ, Nanning SongJing TianLun Biological Technology Co., LTD, Nanning, China) (15–18).

### Morphological Observations of Spermatozoa

Boar sperm was stained with Eosin Y (1%) (15–17). Spermatozoa abnormalities were then viewed using an optical microscope and were classified into head or tail morphological abnormalities: two heads, two tails, blunt hooks, and short tails. The examinations were repeated three times, and 500 spermatozoa per animal were scored.

### Boar Feces Microbiota Sequencing

Total genome DNA from feces was extracted using E.Z.N.A.R Stool DNA Kit (Omega Biotek Inc, USA). The V3-V4 region of the bacterial 16S rRNA gene was amplified using a specific primer (338F, 5'-ACTCCTACGGGAGGCAGCAG-3'; 806R, 5'-GGACTACHVGGGTWTCTAAT-3'). Then the library was sequenced on the Illumina HiSeq 2500 platform and 300 bp paired-end reads were generated at the Novo gene. The sequences were analyzed and assigned to operational taxonomic units (OTUs; 97% identity). OUT abundance information was normalized using a standard of sequence number corresponding to the sample with the least sequences (12, 13).

### Plasma Metabolites Determined by LC-MS/MS

The metabolites were detected as reported in our early study (19). Briefly, boar plasma was collected and maintained at −80°C. LC-MS/MS analysis with ACQUITY UPLC and AB Sciex Triple TOF 5600 (LC/MS) was applied (19). Before LC-MS/MS analysis, the serum samples (100 μl) were precipitated with 10 μl methanol (0.3 mg/ml L-2-Chlorophe) on ice and processed to remove proteins. All samples were then centrifuged at 13,000 g for 10 min at 4°C and the supernatants were injected onto a ACQUITY UPLC HSS T3 column, which was at a flow rate of 0.35 ml/min with 2 μl mobile phase. MS analyses were conducted using electrospray ionization in the positive and negative ion models. Using full scan analysis. Progenesis QI v. 2.3 (Nonlinear Dynamics, Newcastle, UK) was applied to normalize the peaks. Human Metabolome Database (HMDB), Lipidmaps (v. 2.3), and METLIN software were applied to qualify the data. Furthermore, the data were analyzed with SIMCA software (v. 14.0, Umetrics, Umeå, Sweden) and KEGG database (https://www.kegg.jp/) was applied for the pathway enrichment analysis (19).

### Detection of Protein Levels and Location in Spermatozoa Using Immunofluorescence Staining

Microscope slides loaded with boar sperm cells were incubated with 2% Triton X-100 for 1 h at room temperature. Then the samples were blocked for non-specific binding by incubating in 1% BSA with 1% goat serum for 30 min at room temperature. After washing, the cells were incubated with appropriate diluted primary antibodies (anti-Catsper, 1:100; anti-p-ERK, 1:100; anti-PKA, 1:100; anti-ZAG, 1:100) at 4°C overnight and then stained with secondary antibodies (Alexa Flour 546 goat-anti-rabbit IgG) at 1:200 dilution in PBS for 30 min at room temperature. Cell nuclei were counterstained with DAPI. All the samples were observed at room temperature using Leica Laser Scanning Confocal Microscope (LEICA TCS SP% II, Germany) (17).

### Determination of Protein Levels by Western Blotting

Proteins was isolated from sperm cells using RIPA buffer containing protease and phosphate inhibitors (Sangong Biotech, Ltd, Shanghai, China). Total protein concentration was measured using BCA kit (Beyotime Institute of Biotechnology, Shanghai, Chain) according to the manufacture's instruction. Fifty microgram protein was loaded onto 10% polyacrylamide gels and transferred to polyvinylidene fluoride (PVDF) PVDF membrane. The membranes were blocked for 1 h at room temperature in TBST buffer containing 5% non-fat milk. Then the membranes were incubated overnight at 4°C with the primary antibodies (Novex® by life technologies, USA) at a dilution of 1:500 prepares in blocking solution. The control samples were incubated with actin antibody. After washed 3 times, the membranes were incubated with secondary donkey anti-goat IgG-HRP (Beyotime Institute of Biotechnology, Shanghai, P.R. China) or goat anti-rabbit IgG-HRP (Novex® by life technologies, USA) for 1 h at room temperature. finally, the blots were imaged following three washes (12, 13, 17).

### Statistical Analysis

All statistical analyses were performed by using the student's *t*-test (SPSS 21 software). Spearman correlation analysis between the relative abundance of gut microbiota and plasma metabolites using GraphPad Prism 7.0. Data are expressed as the mean ± SEM. *P*-value < 0.05 was considered significant.

### Data Availability

The microbiota raw sequencing data generated in this study has been uploaded to the NCBI SRA database with the accession number PRJNA779574.

## Results

### Effects of HT on Semen Quality

The semen quality was analyzed by using the semen obtained from the last two collections before the end of the experiment. Compared to control group, dietary supplementation of HT tended to increase sperm motility and semen volume/ejaculation even though not significantly (Figures 1B,C). However, HT did not alter sperm concentration or abnormal rate (Figures 1D,E).

### Effects of HT on the Protein Expression of Important Genes Related to Sperm Quality

**Figure 2:**
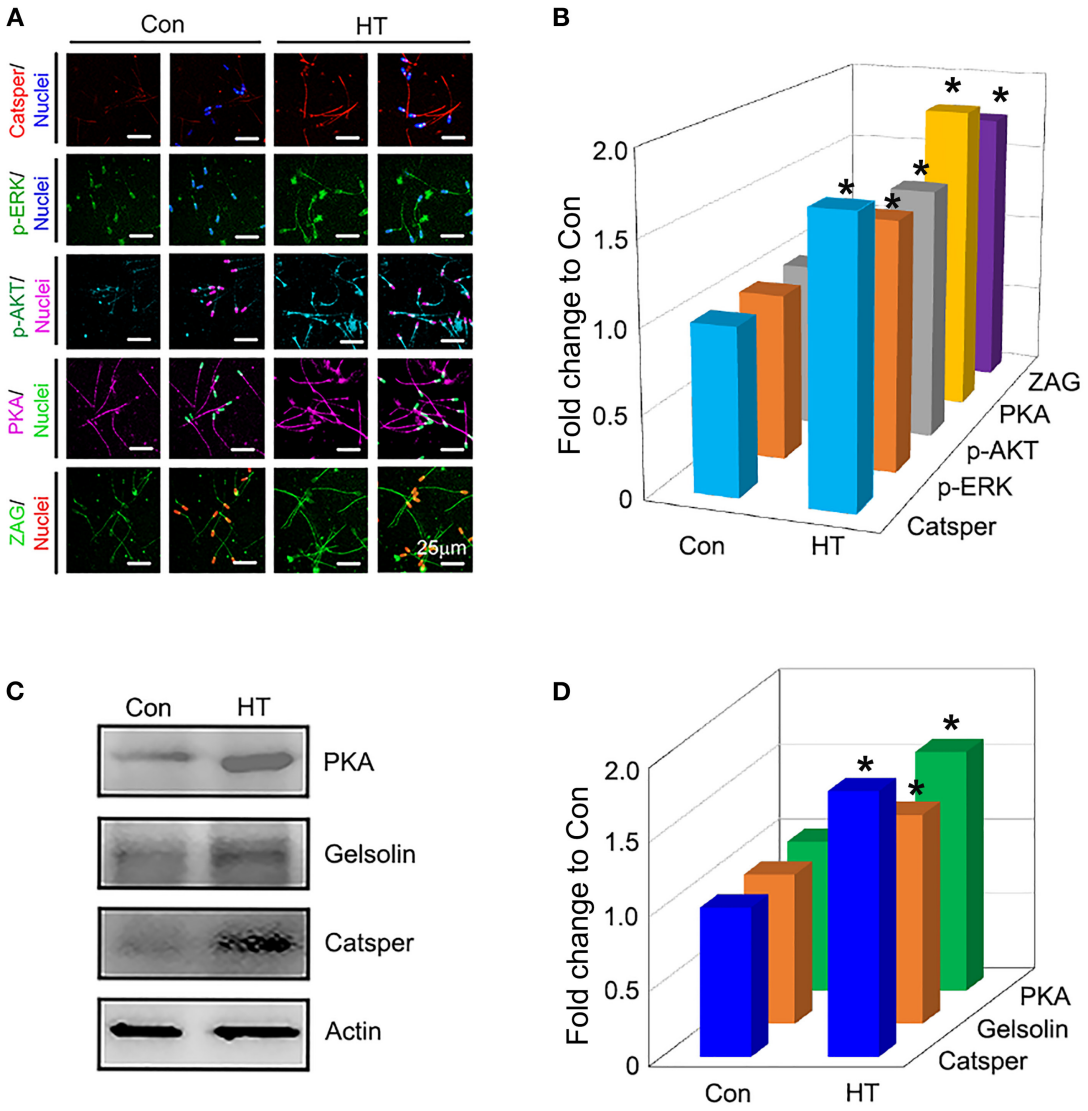
Effects of HT on the protein expression of important genes related to sperm quality. **(A)** Immunofluorescence staining (IHF) of Catsper, p-ERK, p-ERK, PKA, and ZAG. **(B)** Quantitative data for IHF staining (Fold change to Con). **(C)** Western blotting (WB) of PKA, Gelsolin, and Catsper. **(D)** Quantitative data for WB staining (Fold change to Con). *P < 0.05.

Compared to control group, dietary supplementation of HT increased the protein levels of important genes related to sperm quality such as *Catsper, p-ERK, PKA, p-AKT*, and *ZAG* by IHF staining analysis (Figures 2A,B; *P* < 0.05). The data were confirmed by the WB analysis of *Catsper, Gelsolin*, and *PKA* (Figures 2C,D; *P* < 0.05) which suggested that HT improved sperm quality by enhancing the protein expression of important genes related to sperm quality.

### Improvement of HT on Fecal Microbiota

The results showed that dietary supplementation of HT had no significant effects on the α-diversity of fecal microbiota, which was characterized by ACE, Chao1, Simpson, and Shannon indexes (Supplementary Figure 1A). There were 1,109 common OTUs for both Con and HT groups (Supplementary Figure 1B). Moreover, the Con group and HT group contained specific 93 and 77 OTUs, respectively (Supplementary Figure 1B).

**Figure 3:**
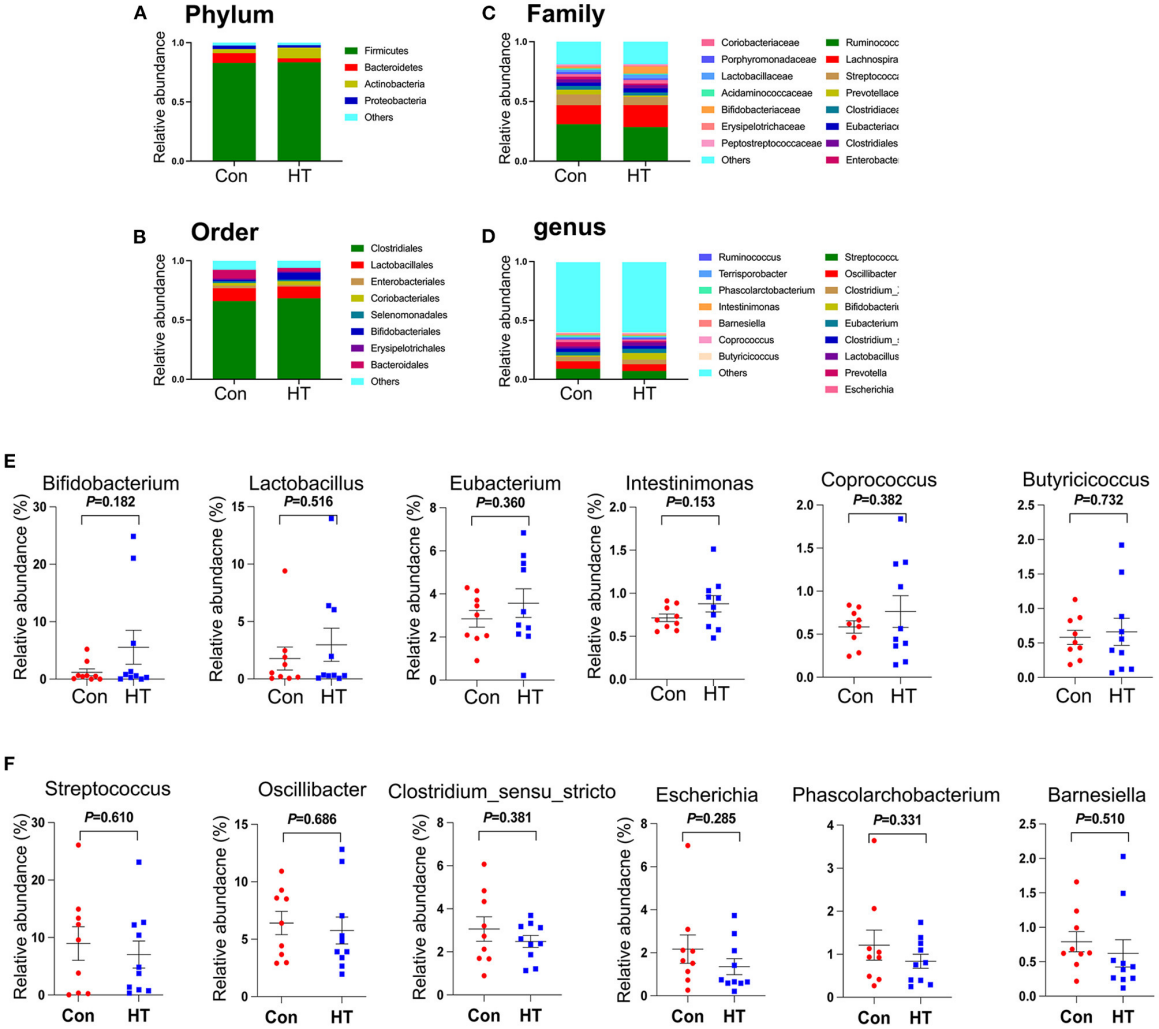
Effects of HT on the fecal microbial composition. **(A)** The relative amount of microbiota in feces at Phylum level. **(B)** The relative amount of microbiota in feces at Order level. **(C)** The relative amount of microbiota in feces at Family level. The relative amount of microbiota in feces at Genus level **(D–F)**. Data were expressed as the mean ± SEM.

The microbial structure was different between HT group and Con group by PCoA analysis (Supplementary Figure 1C). At the phylum level, HT tended to reduce the relative abundance of *Bacteroidetes* and to increase the relative abundance of *Actinobacteria* (Figure 3A; Supplementary Figure 1E). At the order level, HT tended to decrease the level of *Bacteroidales* and to increase the relative abundance of *Bifidobacteriales* (Figure 3B; Supplementary Figure 1F). Moreover, at the family level, HT had the tendency to decrease the relative abundance of *Prevotellaceae* (Figure 3C; Supplementary Figure 1G). At the genus level, HT tended to increase the relative abundance of “beneficial microbes” *Bifidobacterium, Lactobacillus, Eubacterium, Intestinimonas, Coprococcus, and Butyricicoccus* (Figures 3D,E; Supplementary Figure 1H), however, to decrease the relative abundance of “harmful microbes” *Streptococcus, Oscillibacter, Clostridium_sensu_stricto, Escherichia, Phascolarctobacterium*, and *Barnesiella* (Figures 3D,F; Supplementary Figure 1H) (12). LDA effect size (LEfSe) analysis of the taxonomic alterations revealed that the content of some microbiota was different between HT group and control group (Supplementary Figure 1D).

### Effects of HT Supplement on the Plasma Metabolites

**Figure 4:**
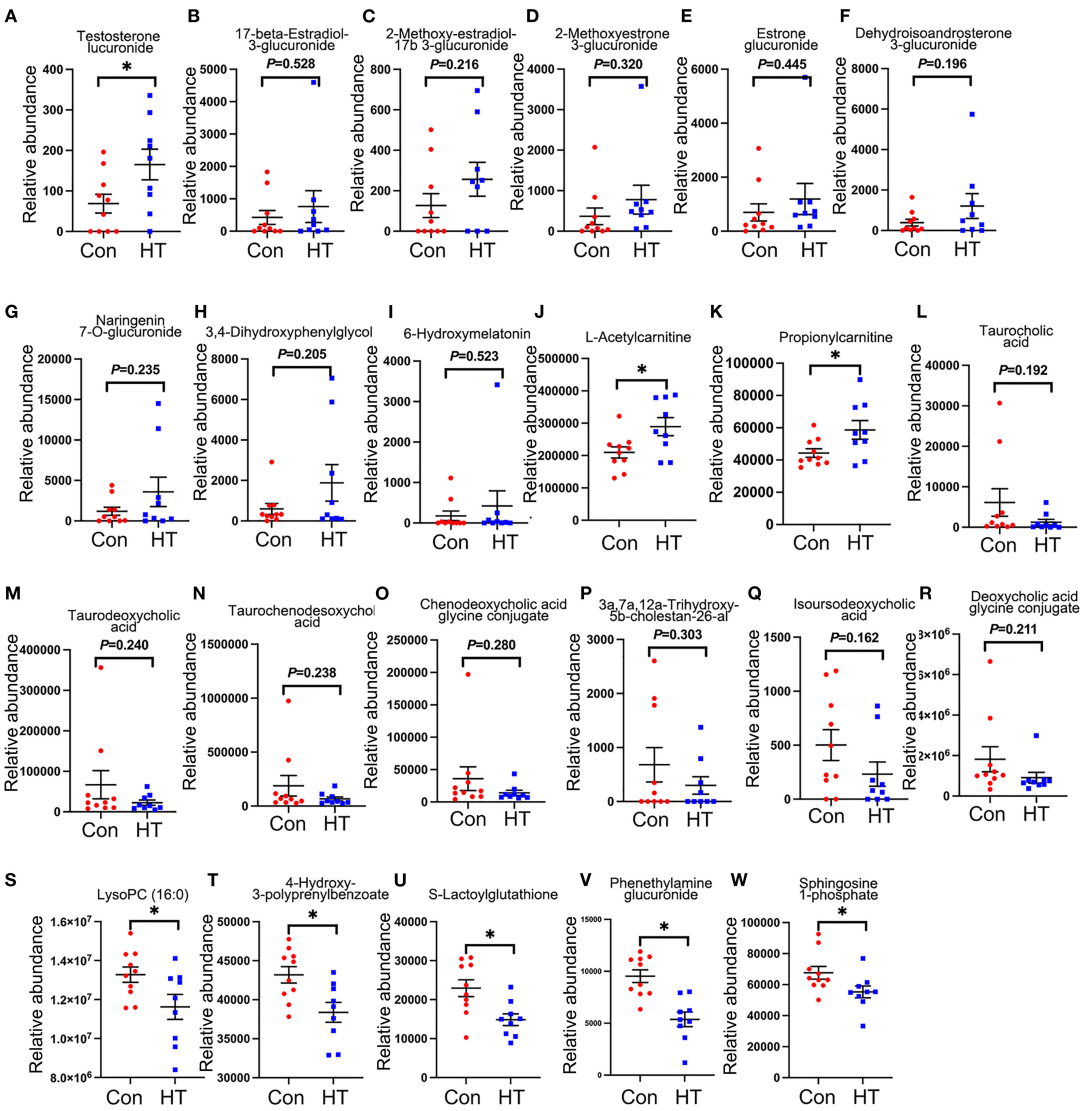
HT-induced alterations in plasma metabolites. **(A–W)** The relative quantification of plasma metabolites. Data were expressed as the mean ± SEM. *P < 0.05.

Compared to control group, HT significantly changed boar plasma metabolites indicated by the plot from the partial least squares discriminant analysis (PLS-DA) (Supplementary Figure 2A). There were 16 significantly changed metabolites between the HT and Con groups (Supplementary Figure 2B). The potential metabolic pathways of the changed metabolites were determined by KEGG pathway analysis. The results showed that the changed metabolites were involved in sphingolipid metabolism, Fc gamma R-mediated phagocytosis, apelin signaling pathway, choline metabolism in cancer, calcium signaling pathway, phospholipase D signaling pathway, tuberculosis, sphingolipid signaling pathway, and insulin resistance signaling pathways (Supplementary Figure 2C). It is very interesting to notice that HT increased plasma level of steroid hormones and their derivatives (Figures 4A–F), especially testosterone glucuronide (Figure 4A). And HT tended to increase the plasma levels of flavonoids: Naringenin 7-O-glucuronide, and 3,4-Dihydroxyphenylglycol (Figures 4G,H). Moreover, it is very interesting that melatonin metabolite 6-hydroxymelatonin, L-Acetylcarnitine and Propionylcarnitine were also elevated by HT in boar plasma although not significantly (Figures 4I–K). However, HT decreased plasma levels of bile acids: taurocholic, taurodeoxycholic acid, taurochenodesoxycholic acid, chenodeoxycholic acid glycine conjugate, 3a,7a,12a-Trihydroxy-5b-cholestan-26-al, isoursodeoxycholic acid, and deoxycholic acid glycine conjugate (Figures 4L–R). Furthermore, HT tended to decrease plasma LysoPC (16:0), 4-Hydroxy-3-polyprenylbenzoate, Phenethylamine glucuronide and Sphingosine 1-phosphate (Figures 4S–W).

### Correlation of Fecal Microbiota, Plasma Metabolites, and/or Sperm Quality

**Figure 5:**
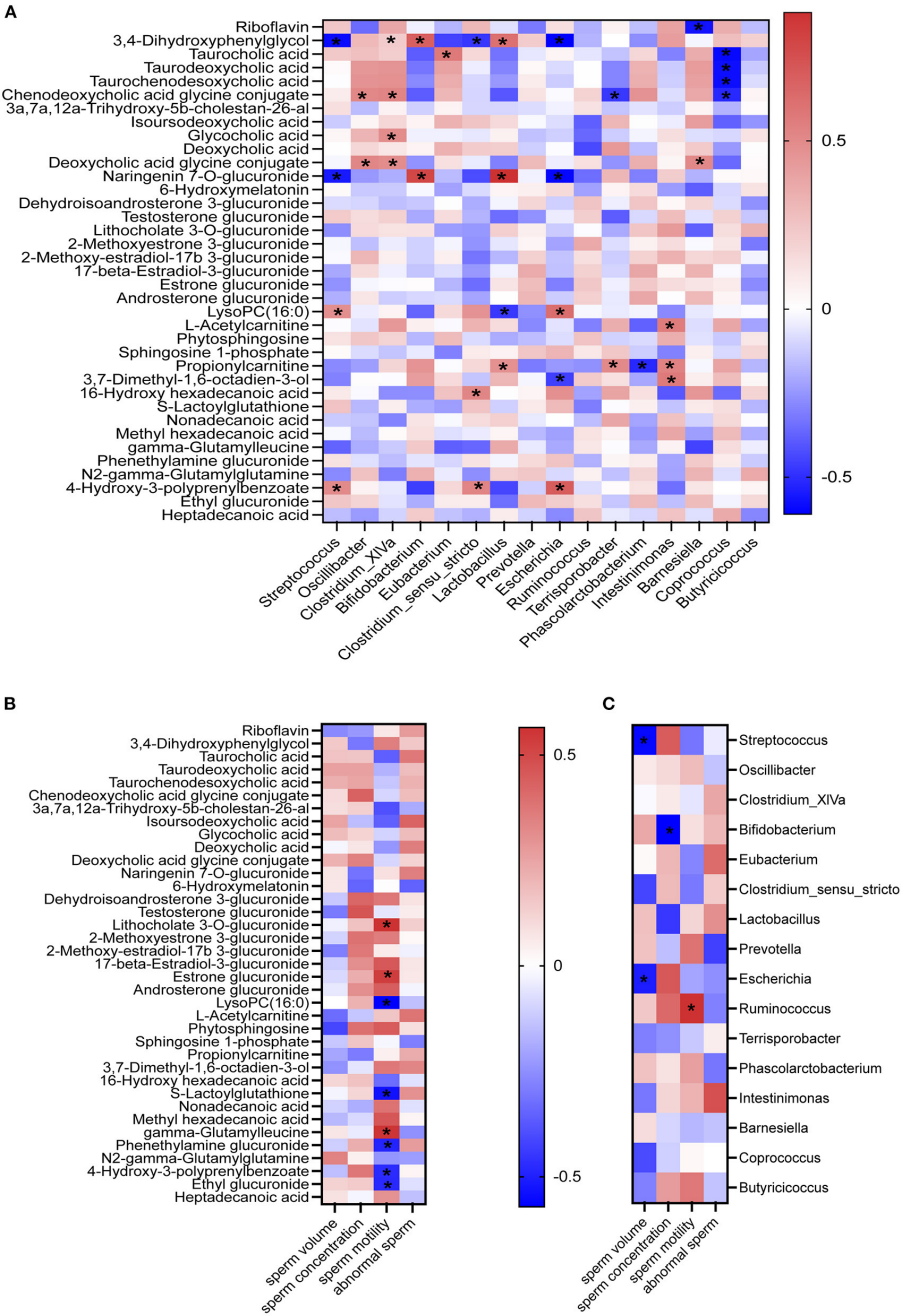
Correlations. Correlations between fecal microbiota and plasma metabolites **(A)**; between plasma metabolites and semen quality parameters **(B)**; and between fecal microbiota and semen quality parameters **(C)** by Pearson correlation analyses. *P < 0.05.

There was very good correlation between fecal microbiota and plasma metabolites, between plasma metabolites and semen parameters, and between fecal microbiota and semen parameters (the average of semen quality of each boar obtained from the last two collections before the end of the experiment) by the spearman correlation analysis (Figure 5). As shown in Figure 5A, the fecal *Lactobacillus* and *Bifidobacterium* were positively correlated with the plasma naringenin 7-O-glucuronide; while *Streptococcus* and *Escherichia* were negatively correlated with the plasma 3,4-dihydroxyphenylglycol and naringenin 7-O-glucuronide; *Coprococcus* was negatively correlated with plasma taurocholic, taurodeoxycholic acid, taurochenodesoxycholic acid, chenodeoxycholic acid glycine conjugate. Plasma LysoPC (16:0), S-lactoylglutathione, phenethylamine glucuronide, 4-hydroxy-3-polyprenylbenzoate, and ethyl glucuronide were negatively correlated with sperm motility (Figure 5B); while plasma gamma-glutamyl leucine, lithocholate 3-O-glucuronide, estrone glucuronide, and gamma-glutamylleucine were positively correlated with sperm motility (Figure 5B). Moreover, there was a significantly positive association between the sperm motility and the fecal *Ruminococcus* and a significantly negative association between the sperm volume and the fecal *Streptococcus* and *Escherichia* (Figure 5C).

### Long-Term Beneficial Effects of HT on Semen Quality

**Figure 6:**
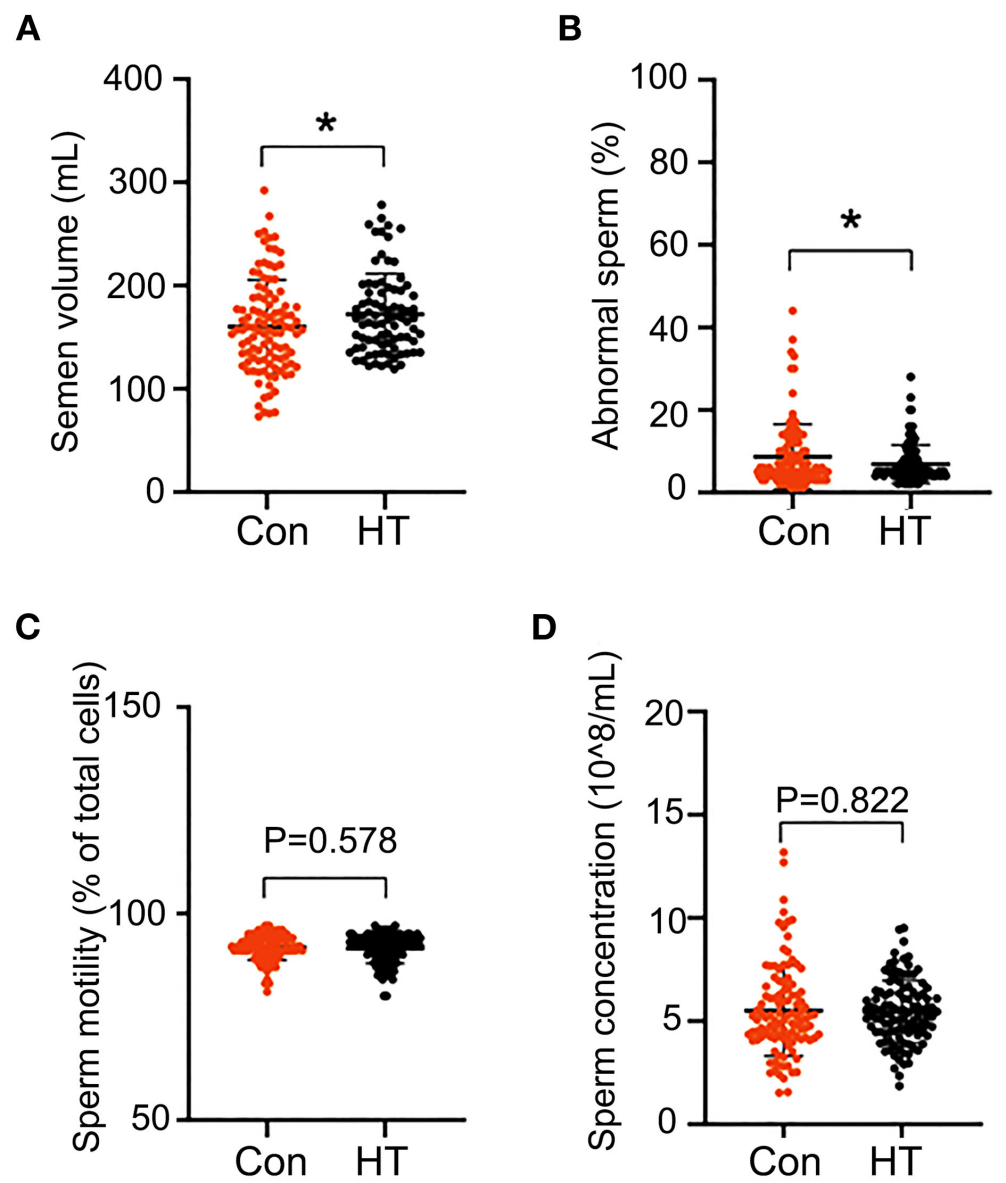
Long-term effects of HT on semen quality. After HT supplementation, semen quality was determined every 5 days for 2 months (no HT supplementation). **(A)** Semen volume. **(B)** Abnormal sperm. **(C)** Sperm motility. **(D)** Sperm concentration. Data were expressed as the mean ± SEM. *P < 0.05.

HT had a long-term beneficial effect on boar semen quality by the increase in the semen volume and the decrease in the percentage of abnormal sperm in the follow-up 2 months analysis (without HT supplementation) (Figures 6A,B). However, HT did not change sperm concentration and motility in the follow-up determination (Figures 6C,D).

## Discussion

Previous studies have reported that dietary supplementation of antioxidants, such as lysine and L-arginine could improve sperm quality in boars (3, 4). Our recent study found that HT had antioxidant effects via modulating gut microbiota and enhancing the expressions of antioxidant enzymes in mice (11). In current investigation, we found that dietary supplementation of HT tended to increase the semen quality, which may be associated with the alterations in gut microbiota and plasma metabolites.

Accumulating evidence suggests that gut microbiota involves in various perspectives of host health (20, 21). Moreover, previous studies have well-established the causal relationship between the gut microbiota and sperm quality in both animal models and humans (1, 12, 13, 22, 23). Polyphenols, including HT, can be metabolized by gut microbiota in the colon and on the other hand HT can modify gut microbiota (24, 25). HT enhanced the relative abundance of *Actinobacteria, Prevotellaceae_UCG-001*, and *Lactobacillus johnsoniiin* in mice (26, 27). Similarly, in current study, we found that HT tended to increase the relative abundance of *Lactobacillus*, which has been shown to have the ability to improve sperm motility in zebrafish (28, 29). Moreover, it has been shown that the increase in the relative abundance of *Bacteroides* and *Prevotella* was associated with higher circulating endotoxin and decreased spermatogenesis (22). HT decreased the relative amount of *Prevotella* and *Bacteroides* in current study. We previously found that alginate oligosaccharides (AOS) could enhance the sperm motility and concentration by the increase in the relative abundance of *Bifidobacteriales* in mice (12). In current study, we found that HT tended to increase the relative abundance of *Bifidobacteriales*. Collectively, our data suggested that HT had the potential to improve spermatogenesis and sperm motility by benefiting gut microbiota.

Our previous study has shown that improved gut microbiota can enhance sperm quality by altering circulating metabolome (12). In current investigation, dietary supplementation of HT altered plasma metabolites by the increase in steroid hormones, antioxidant molecules, and many others. Testosterone and its derivatives play vital roles in spermatogenesis which were increased by HT in boar plasma. L-carnitine plays an important role in improving sperm motility and has been used to treat infertility due to its strong anti-oxidant and anti-inflammatory effects in men and animal models (30–33). It has been shown that epididymis has high level of L-carnitine (32). Interestingly, in current investigation, HT enhanced the plasma level of propionyl-L-carnitine, which is a derivate of L-carnitine and can enhance cellular content of L-carnitine (34). Additionally, in this study, we found that HT enhanced the plasma levels of flavonoids and riboflavin. It has been shown that flavonoids and flavanone metabolites, such as naringenin 7-O-glucuronide and 3,4-dihydroxyphenylglycol, had powerful antioxidant ability, thus they may contribute to improved sperm quality and function (35–38). 6-Hydroxymelatonin (increased by HT) is one of the metabolites of melatonin and has been shown to have strong antioxidant activity (39). Thus, we speculate that HT might improve sperm quality by enhancing the antioxidant capacity of boars. Furthermore, various studies have reported that bile acids can cause oxidative stress by promoting the production of oxygen free radicals from mitochondria (40). Moreover, bile acids contribute to infertility by activating farnesoid X receptor and G-protein-coupled bile acid receptor expressed in sperm, which then influence glucose and lipid metabolism and lead to abnormal sperm (41, 42). In current study, plasma levels of several bile acids and their derivates were decreased by HT. Furthermore, there was a positive correlation between the plasma level of propionyl-L-carnitine and the relative abundance of *Lactobacillus*. Gut microbiota, including *Lactobacillus*, regulates the L-carnitine metabolism (43, 44). Collectively, these data demonstrated that HT might have the potential to improve plasma metabolome to benefit spermatogenesis.

Indeed, HT improved spermatogenesis by the increase in the protein levels of important genes (*PKA, p-AKT, p-ERK, Catsper, ZAG*, and *Gelsolin*) for spermatogenesis and sperm quality. Cation channel of sperm (Catsper) is the primary spermatozoan calcium ion channel and plays an essential role in male fertility via modulating sperm tail calcium entry and sperm hyperactivated motility (45). It has been reported that the cyclic adenosine monophosphate (cAMP) dependent protein kinase (protein kinase A, PKA), AKT, and ERK signaling are involved in modulating the sperm maturation, capacitation, and motility (46, 47). Zn-alpha2-glycoprotein (ZAG) can promote sperm motility via cAMP/PKA signaling pathway (48). Notably, in current study, HT increased the protein levels of these genes which suggested HT enhancing spermatogenesis.

The underlying mechanisms of HT improving semen quality is that it benefits gut microbiota to improve plamsa metabolites then to enhance spermatogenesis and semen quality. The data were confirmed by the follow-up long-term analysis. After the feeding period, all the boars were fed a basal diet (without HT supplementation) and semen samples were collected and analyzed every 5 days for 2 months. HT increased semen volume while decreased the percentage of abnormal sperm in the follow-up 2 months. In summary, HT improves boar semen quality via the benefiting on gut microbiota and plamsa metabolome. HT may be applied as dietary additive to improve boar semen quality in swine industry.

## Data Availability Statement

The datasets presented in this study can be found in online repositories. The names of the repository/repositories and accession number(s) can be found in the article/Supplementary Material.

## Ethics Statement

The animal study was reviewed and approved by the Animal Care and Use Committee of the Institute of Animal Sciences of Chinese Academy of Agricultural Sciences (IAS2021-67).

## Author Contributions

YZha and HZ designed the experiment. HH, RZ, YZho, BX, LC, LL, HS, JT, and FT conducted the experiment and analyzed the data. HZ and YZha wrote and edited the manuscript. All authors contributed to the article and approved the submitted version.

## Funding

This research was supported by the Agricultural Science and Technology Innovation Program (CAAS-ZDRW202006-02, ASTIPIAS07) and the State Key Laboratory of Animal Nutrition (2004DA125184G2102).

## Conflict of Interest

HS and JT were employed by company YangXiang Joint Stock Company. FT was employed by company Hangzhou Viablife Biotech Co., Ltd. The remaining authors declare that the research was conducted in the absence of any commercial or financial relationships that could be construed as a potential conflict of interest. The handling editor declared a shared affiliation with the authors HH, RZ, YZ, BX, LC, YJ, and LL at the time of review.

## Publisher's Note

All claims expressed in this article are solely those of the authors and do not necessarily represent those of their affiliated organizations, or those of the publisher, the editors and the reviewers. Any product that may be evaluated in this article, or claim that may be made by its manufacturer, is not guaranteed or endorsed by the publisher.

## Supplementary Material

Supplementary Figure 1 Effects of HT on the fecal microbial diversity. **(A)** α-diversity with chao1, ACE, Shannon, and Simpson indexes. **(B)** Venn diagram of OUT. **(C)** PCoA of OUT. **(D)** Phylum level. **(E)** Order level. **(F)** Family level; **(G)** Genus level.

Supplementary Figure 2 Effects of HT on plasma metabolites. **(A)** OPLS-DA. **(B)** Heatmap of altered serum metabolites. **(C)** KEGG enriched pathways of altered plasma metabolites.

Supplementary Table 1 Primary antibody information.

Supplementary Data Set 1 Plasma metabolites.

## References

[1] Guo L Wu Y Wang C Wei H Tan J Sun H. Gut microbiological disorders reduce semen utilization rate in duroc boars. Front Microbiol. (2020) 11:581926. 10.3389/fmicb.2020.581926 33133051 PMC7578402

[2] Wu YH Lai W Liu ZH Wei HK Zhou YF Tan JJ. Serum and seminal plasma element concentrations in relation to semen quality in Duroc boars. Biol Trace Elem Res. (2019) 189:85–94. 10.1007/s12011-018-1459-y 30069693

[3] Chen JQ Li YS Li ZJ Lu HX Zhu PQ Li CM. Dietary l-arginine supplementation improves semen quality and libido of boars under high ambient temperature. Animal. (2018) 12:1611–20. 10.1017/S1751731117003147 29198215

[4] Dong HJ Wu D Xu SY Li Q Fang ZF Che LQ. Effect of dietary supplementation with amino acids on boar sperm quality and fertility. Anim Reprod Sci. (2016) 172:182–9. 10.1016/j.anireprosci.2016.08.003 27509874

[5] He Y Li D Zhang W Tian X Pang W Du R. Boar sperm quality and oxidative status as affected by rosmarinic acid at 17 °C. Trop Anim Health Prod. (2020) 52:2169–77. 10.1007/s11250-020-02246-1 32124183

[6] Ren Z Shaoyong W Li Q Ma L Xiao J Jiao J. Effects of Isatis root polysaccharide on boar sperm quality during liquid storage and in vitro fertilization. Anim Reprod Sci. (2019) 210:106178. 10.1016/j.anireprosci.2019.106178 31635774

[7] Namula Z Sato Y Kodama R Morinaga K Luu VV Taniguchi M. Motility and fertility of boar semen after liquid preservation at 5°C for more than 2 weeks. Anim Sci J. (2013) 84:600–6. 10.1111/asj.12049 23607795

[8] Bertelli M Kiani AK Paolacci S Manara E Kurti D Dhuli K. Hydroxytyrosol: a natural compound with promising pharmacological activities. J Biotechnol. (2020) 309:29–33. 10.1016/j.jbiotec.2019.12.016 31884046

[9] Karković Marković A Torić J Barbarić M Jakobušić Brala C. Hydroxytyrosol, tyrosol and derivatives and their potential effects on human health. Molecules. (2019) 24:2001. 10.3390/molecules24102001 31137753 PMC6571782

[10] Wei J Wang S Pei D Qu L Li Y Chen J. Antibacterial activity of hydroxytyrosol acetate from olive leaves (Olea Europaea L.). Nat Prod Res. (2018) 32:1967–70. 10.1080/14786419.2017.1356830 28768425

[11] Wang M Zhang S Zhong R Wan F Chen L Liu L. Olive fruit extracts supplement improve antioxidant capacity via altering colonic microbiota composition in mice. Front Nutr. (2021) 8:645099. 10.3389/fnut.2021.645099 33889594 PMC8055859

[12] Zhang P Feng Y Li L Ge W Yu S Hao Y. Improvement in sperm quality and spermatogenesis following faecal microbiota transplantation from alginate oligosaccharide dosed mice. Gut. (2021) 70:222–5. 10.1136/gutjnl-2020-320992 32303608 PMC7788261

[13] Zhao Y Zhang P Ge W Feng Y Li L Sun Z. Alginate oligosaccharides improve germ cell development and testicular microenvironment to rescue busulfan disrupted spermatogenesis. Theranostics. (2020) 10:3308–24. 10.7150/thno.43189 32194870 PMC7053202

[14] Wu YH Guo LL Liu ZH Wei HK Zhou YF Tan JJ. Microelements in seminal and serum plasma are associated with fresh semen quality in Yorkshire boars. Theriogenology. (2019) 132:88–94. 10.1016/j.theriogenology.2019.04.002 31004878

[15] Zhang W Zhao Y Zhang P Hao Y Yu S Min L. Decrease in male mouse fertility by hydrogen sulfide and/or ammonia can be inheritable. Chemosphere. (2018) 194:147–57. 10.1016/j.chemosphere.2017.11.164 29202267

[16] Zhang P Zhao Y Zhang H Liu J Feng Y Yin S. Low dose chlorothalonil impairs mouse spermatogenesis through the intertwining of estrogen receptor pathways with histone and DNA methylation. Chemosphere. (2019) 230:384–95. 10.1016/j.chemosphere.2019.05.029 31112861

[17] Zhao Y Zhang W Liu X Zhang P Hao Y Li L. Hydrogen sulfide and/or ammonia reduces spermatozoa motility through AMPK/AKT related pathways. Sci Rep. (2016) 6:37884. 10.1038/srep37884 27883089 PMC5121643

[18] WHO. WHO Laboratory Manual for the Examination and Processing of Human Semen. 5th ed. Cambridge: Cambridge University Press (2010).

[19] Zhang P Liu J Xiong B Zhang C Kang B Gao Y. Microbiota from alginate oligosaccharide dosed mice successfully mitigated small intestinal mucositis. Microbiome. (2020) 8:112. 10.1186/s40168-020-00886-x 32711581 PMC7382812

[20] Han H Jiang Y Wang M Melaku M Liu L Zhao Y. Intestinal dysbiosis in nonalcoholic fatty liver disease (NAFLD): focusing on the gut-liver axis. Crit Rev Food Sci Nutr. (2021) 1–18. 10.1080/10408398.2021.1966738 34404276

[21] Han H Yi B Zhong R Wang M Zhang S Ma J. From gut microbiota to host appetite: gut microbiota-derived metabolites as key regulators. Microbiome. (2021) 9:162. 10.1186/s40168-021-01093-y 34284827 PMC8293578

[22] Ding N Zhang X Zhang XD Jing J Liu SS Mu YP. Impairment of spermatogenesis and sperm motility by the high-fat diet-induced dysbiosis of gut microbes. Gut. (2020) 69:1608–19. 10.1136/gutjnl-2019-319127 31900292 PMC7456731

[23] Lundy SD Sangwan N Parekh NV Selvam MKP Gupta S McCaffrey P. Functional and taxonomic dysbiosis of the gut, urine, and semen microbiomes in male infertility. Eur Urol. (2021) 79:826–36. 10.1016/j.eururo.2021.01.014 33573862

[24] Tuck KL Freeman MP Hayball PJ Stretch GL Stupans I. The in vivo fate of hydroxytyrosol and tyrosol, antioxidant phenolic constituents of olive oil, after intravenous and oral dosing of labeled compounds to rats. J Nutr. (2001) 131:1993–6. 10.1093/jn/131.7.1993 11435519

[25] Visioli F Galli C Bornet F Mattei A Patelli R Galli G. Olive oil phenolics are dose-dependently absorbed in humans. FEBS Lett. (2000) 468:159–60. 10.1016/S0014-5793(00)01216-3 10692578

[26] Liu Z Wang N Ma Y Wen D. Hydroxytyrosol improves obesity and insulin resistance by modulating gut microbiota in high-fat diet-induced obese mice. Front Microbiol. (2019) 10:390. 10.3389/fmicb.2019.00390 30886607 PMC6410680

[27] Wang N Ma Y Liu Z Liu L Yang K Wei Y. Hydroxytyrosol prevents PM(2.5)-induced adiposity and insulin resistance by restraining oxidative stress related NF-κB pathway and modulation of gut microbiota in a murine model. Free Radic Biol Med. (2019) 141:393–407. 10.1016/j.freeradbiomed.2019.07.002 31279968

[28] Valcarce DG Riesco MF Martínez-Vázquez JM Robles V. Diet supplemented with antioxidant and anti-inflammatory probiotics improves sperm quality after only one spermatogenic cycle in zebrafish model. Nutrients. (2019) 11:843. 10.3390/nu11040843 31013929 PMC6549425

[29] Valcarce DG Riesco MF Martínez-Vázquez JM Robles V. Long exposure to a diet supplemented with antioxidant and anti-inflammatory probiotics improves sperm quality and progeny survival in the zebrafish model. Biomolecules. (2019) 9:338. 10.3390/biom9080338 31382562 PMC6724062

[30] Abd-Elrazek AM Ahmed-Farid OAH. Protective effect of L-carnitine and L-arginine against busulfan-induced oligospermia in adult rat. Andrologia. (2018) 50:1–8. 10.1111/and.12806 28444774

[31] Micic S Lalic N Djordjevic D Bojanic N Bogavac-Stanojevic N Busetto GM. Double-blind, randomised, placebo-controlled trial on the effect of L-carnitine and L-acetylcarnitine on sperm parameters in men with idiopathic oligoasthenozoospermia. Andrologia. (2019) 51:e13267. 10.1111/and.13267 30873633 PMC6850469

[32] Mongioi L Calogero AE Vicari E Condorelli RA Russo GI Privitera S. The role of carnitine in male infertility. Andrology. (2016) 4:800–7. 10.1111/andr.12191 27152678

[33] Reuter SE Evans AM. Carnitine and acylcarnitines: pharmacokinetic, pharmacological and clinical aspects. Clin Pharmacokinet. (2012) 51:553–72. 10.1007/BF03261931 22804748

[34] Ferrari R Merli E Cicchitelli G Mele D Fucili A Ceconi C. Therapeutic effects of L-carnitine and propionyl-L-carnitine on cardiovascular diseases: a review. Ann N Y Acad Sci. (2004) 1033:79–91. 10.1196/annals.1320.007 15591005

[35] Anacleto SL Milenkovic D Kroon PA Needs PW Lajolo FM Hassimotto NMA. Citrus flavanone metabolites protect pancreatic-β cells under oxidative stress induced by cholesterol. Food Funct. (2020) 11:8612–24. 10.1039/D0FO01839B 32959863

[36] Kuang W Zhang J Lan Z Deepak R Liu C Ma Z. SLC22A14 is a mitochondrial riboflavin transporter required for sperm oxidative phosphorylation and male fertility. Cell Rep. (2021) 35:109025. 10.1016/j.celrep.2021.109025 33882315 PMC8065176

[37] Saedisomeolia A Ashoori M. Riboflavin in human health: a review of current evidences. Adv Food Nutr Res. (2018) 83:57–81. 10.1016/bs.afnr.2017.11.002 29477226

[38] Tvrdá E Debacker M Duračka M Kováč J Bučko O. Quercetin and naringenin provide functional and antioxidant protection to stored boar semen. Animals. (2020) 10:1930. 10.3390/ani10101930 33096604 PMC7589831

[39] Maharaj DS Walker RB Glass BD Daya S. 6-Hydroxymelatonin protects against cyanide induced oxidative stress in rat brain homogenates. J Chem Neuroanat. (2003) 26:103–7. 10.1016/S0891-0618(03)00034-6 14599659

[40] Bomzon A Holt S Moore K. Bile acids, oxidative stress, and renal function in biliary obstruction. Semin Nephrol. (1997) 17:549–62. 9353865

[41] Baptissart M Vega A Martinot E Pommier AJ Houten SM Marceau G. Bile acids alter male fertility through G-protein-coupled bile acid receptor 1 signaling pathways in mice. Hepatology. (2014) 60:1054–65. 10.1002/hep.27204 24798773

[42] Malivindi R Santoro M De Rose D Panza S Gervasi S Rago V. Activated-farnesoid X receptor (FXR) expressed in human sperm alters its fertilising ability. Reproduction. (2018) 156:249–59. 10.1530/REP-18-0203 29921626

[43] Kim M Kim M Kang M Yoo HJ Kim MS Ahn YT. Effects of weight loss using supplementation with *Lactobacillus* strains on body fat and medium-chain acylcarnitines in overweight individuals. Food Funct. (2017) 8:250–61. 10.1039/C6FO00993J 28001147

[44] Ussher JR Lopaschuk GD Arduini A. Gut microbiota metabolism of L-carnitine and cardiovascular risk. Atherosclerosis. (2013) 231:456–61. 10.1016/j.atherosclerosis.2013.10.013 24267266

[45] Lishko PV Kirichok Y Ren D Navarro B Chung JJ Clapham DE. The control of male fertility by spermatozoan ion channels. Annu Rev Physiol. (2012) 74:453–75. 10.1146/annurev-physiol-020911-153258 22017176 PMC3914660

[46] Baro Graf C Ritagliati C Stival C Luque GM Gentile I Buffone MG. Everything you ever wanted to know about PKA regulation and its involvement in mammalian sperm capacitation. Mol Cell Endocrinol. (2020) 518:110992. 10.1016/j.mce.2020.110992 32853743

[47] Li X Luo T Li H Yan N. Sphingomyelin synthase 2 participate in the regulation of sperm motility and apoptosis. Molecules. (2020) 25:4231. 10.3390/molecules25184231 32942681 PMC7570487

[48] Qu F Ying X Guo W Guo Q Chen G Liu Y. The role of Zn-alpha2 glycoprotein in sperm motility is mediated by changes in cyclic AMP. Reproduction. (2007) 134:569–76. 10.1530/REP-07-0145 17890292

