# Supplementary figures and images for: Hydroxytyrosol Benefits Boar Semen Quality via Improving Gut Microbiota and Blood Metabolome
Source: Front Nutr. 2022 Jan 17;8:815922. doi: 10.3389/fnut.2021.815922 (PMC8802763; doi:10.3389/fnut.2021.815922)

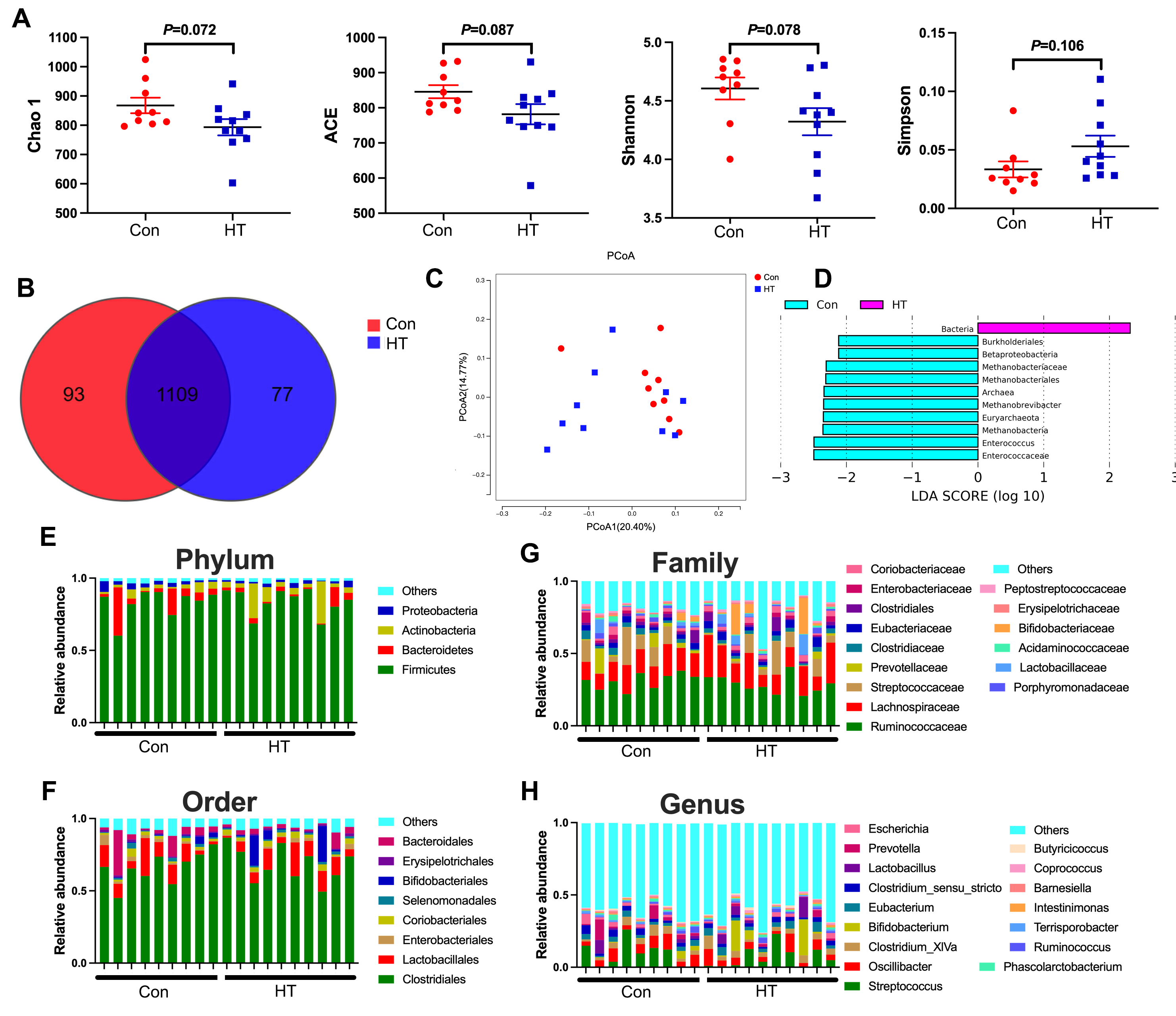

Supplement: Supplementary Figure 1 — Effects of HT on the fecal microbial diversity. (A) α-diversity with chao1, ACE, Shannon, and Simpson indexes. (B) Venn diagram of OUT. (C) PCoA of OUT. (D) Phylum level. (E) Order level. (F) Family level; (G) Genus level. [file Image_1.TIF]

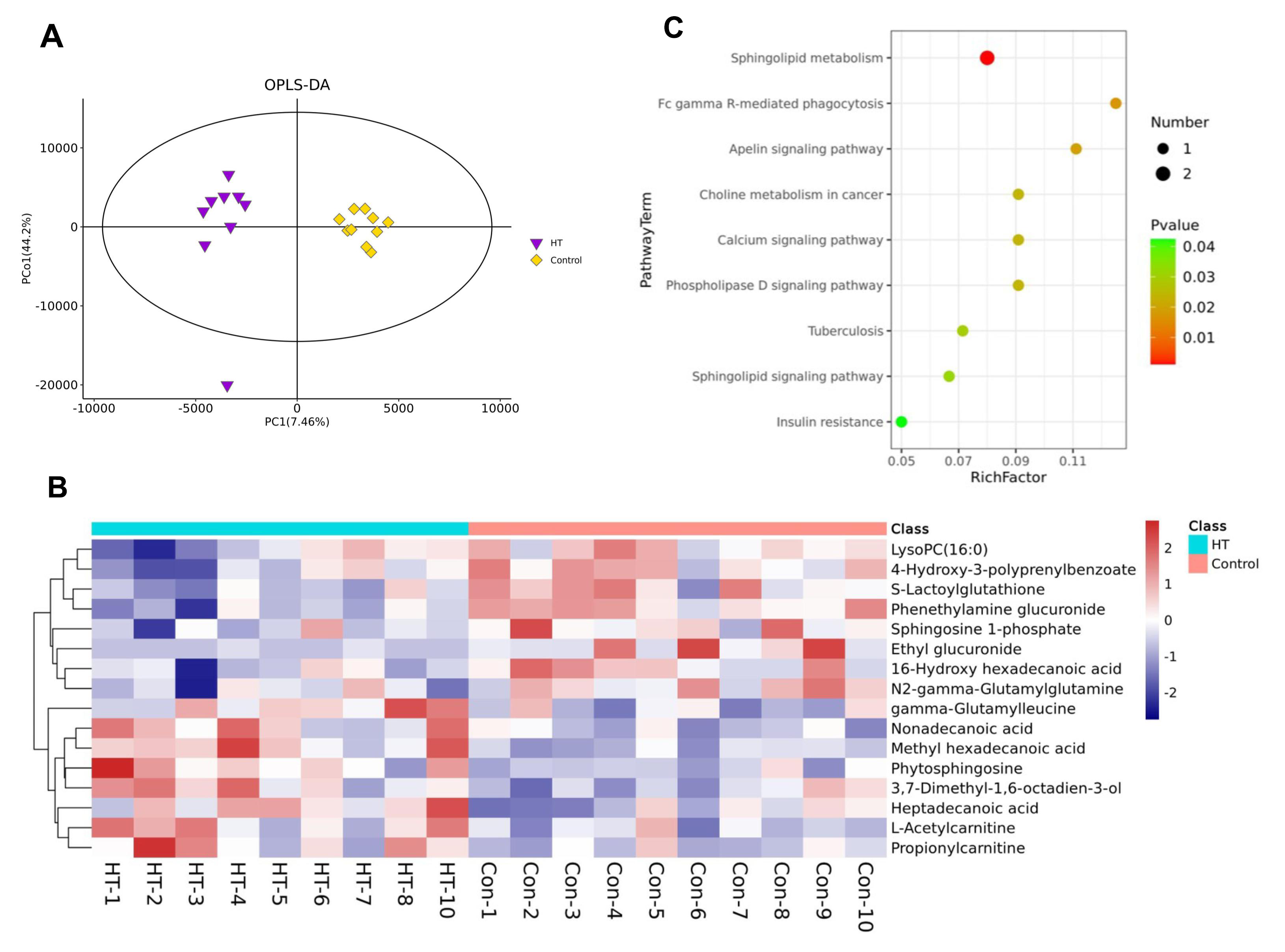

Supplement: Supplementary Figure 2 — Effects of HT on plasma metabolites. (A) OPLS-DA. (B) Heatmap of altered serum metabolites. (C) KEGG enriched pathways of altered plasma metabolites. [file Image_2.TIF]
