# Supplementary material for: Hydroxytyrosol Benefits Boar Semen Quality via Improving Gut Microbiota and Blood Metabolome
Source: Front Nutr. 2022 Jan 17;8:815922. doi: 10.3389/fnut.2021.815922 (PMC8802763; doi:10.3389/fnut.2021.815922)
Supplement: Supplementary Table 1 — Primary antibody information. [file Table_1.docx]

**Table S1.** Primary antibody information

| **Gene symbol** | **Name** | **Cat. #** | **Predicted size** | **Source (Animal)** | **Company** |
| --- | --- | --- | --- | --- | --- |
| Actin | actin | Ab3280 | 42kDa | Rabbit (polyclonal) | Abcam |
| PKA | cAMP dependent protein kinase alpha catalytic subunit | bs-0520R | 40kd | Rabbit (polyclonal) | Beijing Biosynthesis Biotechnology CO. |
| P-ERK | phospho-Erk1 (Thr202 + Tyr204) | bs-1645R | 43kDa | Rabbit (polyclonal) | Beijing Biosynthesis Biotechnology CO. |
| ZAG | Zinc Alpha 2 Glycoprotein | bs-19382R | 32kDa | Rabbit | Beijing Biosynthesis Biotechnology CO. |
| CATSPER | CATSPER | bs-23326R | 90kDa | Rabbit | Beijing Biosynthesis Biotechnology CO. |
| Gelsolin | Gelsolin | bs-1160R | 80kDa | Rabbit | Beijing Biosynthesis Biotechnology CO. |
| p-PI3K | phosphorylated Phosphoinositide 3-kinase | bs-5571R | 80kd | Rabbit (polyclonal) | Beijing Biosynthesis Biotechnology CO. |
| p-AKT | phosphorylated AKT | bs-2720R | 56kd | Rabbit (polyclonal) | Beijing Biosynthesis Biotechnology CO. |
